# Psychological factors associated with vaccination hesitancy: an observational study of patients hospitalized for COVID-19 in a later phase of the pandemic in Italy

**DOI:** 10.3389/fpsyt.2023.1272959

**Published:** 2023-10-19

**Authors:** Carla Comacchio, Maddalena Cesco, Rosita Martinelli, Marco Garzitto, Rita Bianchi, Nicola Innocente, Emanuela Sozio, Carlo Tascini, Matteo Balestrieri, Marco Colizzi

**Affiliations:** ^1^Unit of Psychiatry, Department of Medicine (DAME), University of Udine, Udine, Italy; ^2^Infectious Diseases Division, Department of Medicine (DAME), University of Udine, Udine, Italy; ^3^Department of Psychosis Studies, Institute of Psychiatry, Psychology and Neuroscience, King’s College London, London, United Kingdom

**Keywords:** mental health, vaccination, vaccination hesitancy, COVID-19, resilience

## Abstract

**Introduction:**

Vaccination against SARS-CoV-2 has been used to reduce the severity of COVID-19 disease and the incidence of new cases. However, a significant proportion of people have shown vaccination hesitancy.

**Methods:**

This study explored psychological factors related to vaccination hesitancy in a sample of Italian COVID-19 patients (*N* = 54), hospitalized during 2021, after vaccines had been made available and while the vaccination campaign was on-going. Consecutive patients, aged 18 or older, admitted to the hospital with a diagnosis of COVID-19 were assessed with a set of standardized measures.

**Results:**

In our sample, 48.1% was not vaccinated and 7.4% died within 6months after hospitalization, with a preponderance of deaths among non-vaccinated patients. Non-vaccinated participants had higher resilience scores at the CD-RISC-10 scale than vaccinated ones (33.6 ± 5.50 vs 28.6 ± 6.61; t40.2=+ 2.94, *p* = 0.005). No statistically significant differences were found between the two groups for any other measures.

**Discussion:**

Higher levels of resilience among non-vaccinated patients may reflect greater identity worth and self-esteem, in turn resulting in a decrease in vaccination likelihood. This finding may have important public health implications, as it indicates that specific psychological aspects, such as resilience, may result in vaccination hesitancy, with implications for hospitalization rates, and thus healthcare costs, as well as loss of lives.

## 1. Introduction

COVID-19 is the name of the disease caused by the new coronavirus SARS-CoV-2, discovered in China in December 2019. It was declared by the World Health Organization (WHO) to be a global pandemic in March 2020 (1). Over 600 million people have suffered from COVID-19 and almost 6 million have died by March 10th, 2023, because of the disease (2). The virus spread very quickly around the world, resulting in the decision to implement restrictive measures by public health services, such as social and physical distancing, travel restrictions, use of personal protective equipment, confinement (quarantine), and hygiene measures. Common symptoms of COVID-19 include a dry cough, fever or chills, shortness of breath or difficulty breathing, muscle or body aches, sore throat, loss of taste or smell, diarrhea, headache, fatigue, nausea or vomiting, and congestion or a runny nose. However, the impact of COVID-10 has not been limited to physical health. The virus itself (direct effect) (3) and the measures applied by institutions (indirect effect) (4) have contributed to worsening the quality of life and mental health of the general population (5), along with a higher risk of relapse in individuals with mental health issues before the start of the pandemic (6). Vaccination against SARS-CoV-2 has been used to reduce the severity of COVID-19 disease and the incidence of new cases, leading to a significant change in the course of the pandemic and eventually relaxing restrictions and confinements. Moreover, it has been helpful to minimize possible permanent adverse health consequences and to avoid collapse of the health care systems. However, the success of a vaccination campaign does not depend only on its efficacy and safety, but also on the level of the vaccine acceptance, that may jeopardize the successful control of an infectious disease (7). Reaching out and vaccinating people who accept vaccination is obviously crucial, however the real challenge is to convince reluctant persons to vaccinate. Another factor that influenced the success of the vaccination campaign is represented by the introduction of certain laws that have contributed to increasing the vaccination rate in certain population groups (8, 9). Analyzing data from 33 countries, a recent systematic review found significant variation in terms of COVID-19 vaccine acceptance, with Italy being one of the countries with the lowest levels (10), possibly causing delays in reaching the target population immunization rate (11). Thus, studies are needed to identify predictors of vaccination hesitancy, including psychological characteristics (12), with the goal of mitigating its detrimental effects on control of infectious disease. The aim of the present study was to evaluate the prevalence of vaccination hesitancy in a cohort of COVID-19 hospitalized patients in Italy during a later phase of the pandemic, when the vaccine was already available, and the vaccination campaign was ongoing. Furthermore, the study aimed at identifying psychological factors associated with vaccination hesitancy.

## 2. Materials and methods

This is an observational study. It was conducted at the University hospital of Udine, a tertiary referral hospital of nearly 1,000 beds serving approximately 350,000 inhabitants, which has been appointed as regional hub for COVID-19 patients. The recruitment started on March 20th 2021 and ended December 31st 2022. All consecutive patients, aged 18 or older, admitted at the Infectious Disease Department with a diagnosis of COVID-19 confirmed by molecular swab, were considered eligible for inclusion. For those patients expressing willingness to participate, written informed consent was obtained before data collection. Since interviews were conducted via telephone, exclusion criteria included being unable to undertake a telephone interview due to any medical or psychological condition. The study was conducted in accordance with the Declaration of Helsinki and approved by the local Ethics Committee (CEUR-2021-OS-19).

### 2.1. Psychological assessment tools

Vaccination status for COVID-19 together with sociodemographic information were collected using an *ad hoc* form. Cognitive status was assessed with a back-translated version of the original English Telephone Interview for Cognitive Status (TICS) developed by Brandt in 1988 (13). TICS score ranges from 1 to 41 and comprises 11 items assessing orientation (personal, temporal, and spatial; score range: 0–12), attention and executive functioning (backward counting, backward calculation, abstraction; range: 0–9), language (naming to description, sentence repetition, and oral comprehension; range: 1–8), and memory (immediate recall, semantic memory; range: 0–12). Resilience was assessed with the Connor–Davidson resilience scale (CD-RISC-10). This is the abridged version of a 25-items self-report scale developed by Connor and Davidson in 2003 (14). It measures characteristics such as persistence, optimism, and confidence in ability to cope, with higher score indicating better resilience (15). Illness perception was assessed with the Brief illness perception questionnaire (IPQ-B), developed by Broadbent in 2006 (16). It is an eight-item scale that has each item rated on a scale from 0 (minimum) to 10 (maximum) and which assesses the emotional and cognitive aspects of an individual’s illness. Each item of this questionnaire examines a dimension of understanding of the disease as follows: 1. Consequences; 2. Timeline; 3. Personal control; 4. Treatment control; 5. Identity; 6. Concern; 7. Illness comprehensibility; 8. Emotions. The IPQ-B also includes a question that is answered by the patient about one’s opinion regarding the cause of illness. Items 1–5 assess the cognitive dimensions which relate to understanding of illness, its causes, and effect of treatment, while items 6 to 8 evaluate the emotional dimensions that relate to emotions such as mood, fear, anxiety, or anger. The total score of illness perception is calculated by inverting the score for items 3, 4 and 7 and added to the score of the other items. The maximum total score is 80 and the minimum total score is 0. A higher score indicates a more threatening view of the patient, while a lower score indicates a more optimistic view of the disease. Family function was assessed with the Family APGAR (17). It is a 5-item questionnaire (with each item rated on a 3-point scale) measuring five constructs: 1. Adaptability; 2. Partnership; 3. Growth; 4. Affection; and 5. Resolve. Scores range from 0 to 10 with higher scores indicating better functionality. Protective and risk factors after a potential traumatic event were assessed with the Global Psychodrama Screen (GPS, version 1.2), developed by the ‘Global Collaboration on Traumatic Stress’ (18). It consists of 22 items, 17 symptom items and 5 risk or protective factors, each to be answered in a yes/no format. The 17 symptom items assess: PTSD symptoms; affective dysregulation and negative self-concept; depression symptoms; anxiety symptoms; dissociation; sleep problems; self-injurious behavior; substance abuse; and other problems (physical, emotional, or social). The five risk or protective items assess other stressful events; social support; traumatic life events in childhood; history of psychiatric treatment; and resilience. The GPS total score is calculated using all 22 items, ranging 0–22. A total symptom score is the sum score of the 17 symptom items (range 0–17). A risk factor score is the sum score of the five risk or protective items (range 0–5). The Mini Locus of Control Scale (MLCS) has been used to assess self-perception of control over external events (19). It consists of 6 items. People were asked to state their level of agreement according to a 4-point scale: totally (4); enough (3); little (2); not at all (1). It comprises three main subscales: 1. Fatalism, the random play of external circumstances (“There are those who are born lucky and those who are not”; “Without the right opportunities, it is difficult to succeed in life”); 2. Hetero-dependence, the influence exerted by the social environment (“My life is controlled mainly by the influence of other people”; “It is others who decide whether you succeed in your life or not”); and 3. Internality, the personal wills capabilities (“People could do so much more, if only they really tried”; “It’s entirely up to me if I can take advantage of the opportunities life gives me”).

### 2.2. Statistical analysis

Categorical measures were summarized with percentage frequencies; mean, standard deviation (SD), and range of variation were reported for continuous measures. Fisher’s exact test was used in cross- tables with categorical measures (also reporting Odd-ratios, OR, with their 95% confidence interval, ci). In between-group comparisons, Welch’s corrected t-test (or Mann–Whitney’s test) or one-way analysis of variance were used with continuous measures (checking homoskedasticity with median-centered Levene’s test). Also, Pearson’s correlations were calculated (reporting estimated correlation with its 95% ci). Finally, multiple linear regression was fitted reporting its coefficient of determination (*R*^2^; also adjusted for the number of covariates) and statistical significance; for predictors, tolerances and standardized coefficient (β) with statistical significance were reported. Pair-wise deletion on missing data was adopted. The statistical significance was set to *p* < 0.050. Analyzes were conducted with R-4.2.3 (R Development Core Team, 2023).

## 3. Results

### 3.1. Study participants

Sociodemographic and clinical characteristics of the sample, including details about vaccination status, are described in Table 1. Considering the overall sample, 48.1% of patients were not vaccinated. 7.4% of participants died within 6 months after hospitalization [3.2 ± 1.78 (1.2, 5.0) months]. It was observed a preponderance of deaths in the non-vaccinated group (16.7%) when compared with the vaccinated group [2.8%; OR = 6.727 (IC 95% 0.495, 376.765)]. No statistically significant differences were observed between participants who have been vaccinated and those who were not for age at hospitalization (U = 276.0, *p* = 0.944), sex [OR = 1.528 (IC 95% 0.360, 7.842)], being single [OR = 1.118 (IC 95% 0.304, 4.044)], living alone [OR = 1.898 (IC 95% 0.385, 9.073)], having a low-level of schooling [OR = 0.468 (IC 95% 0.092, 1.948)], working [OR = 0.705 (IC 95% 0.175, 2.612)], having physical comorbidities [OR = 1.000 (IC 95% 0.181, 7.039)], being under psychopharmacological treatment [OR = 2.699 (IC 95% 0.269, 137.102)], and duration of hospitalization (*t*_27.4_ = +0.23, *p* = 0.821).

**Table 1:** Sample description and vaccination status.

	% or Mean ± SD [min, Max]
Number of observations	54
	% or Mean ± SD [min, Max]
Sex	*Female*: 27.8%
Age at hospitalization	62.4 ± 12.58 [27, 85]
Marital status	*Single*: 22.2%
	*Couple/Married*: 57.4%
	*Separated/Divorced*: 14.8%
	*Widower/Widow*: 5.6%
Living situation	*Lives alone*: 20.4%
	*Lives with family*: 74.1%
	*Other living situation*: 5.6%
Schooling	*Primary*: 7.7%
	*Middle*: 25.0%
	*High*: 55.8%
	*Degree*: 11.5%
Work situation	*Retired*: 48.1%
	*Employed*: 38.9%
	*Unemployed*: 9.3%
	*Housewife*: 3.7%
Has physical comorbidity	83.3%
Psychopharmacological therapy	*No*: 88.9%
	*Sleeping medication*: 7.4%
	*Antidepressants*: 1.9%
	*Other medication*: 1.9%
Days of hospitalization	17.8 ± 17.98 [2, 84]
Vaccination status	*Before hospitalization*: 51.9%
	*After hospitalization*: 14.8%
	*Never*: 33.3%
Details	*Single dose*: 7.4%
	*Two doses*: 20.4%
	*Three doses*: 38.9%

Max, Maximum observed value; min, minimum observed value; SD, Standard Deviation.

### 3.2. Psychological assessments

**Figure 1:**
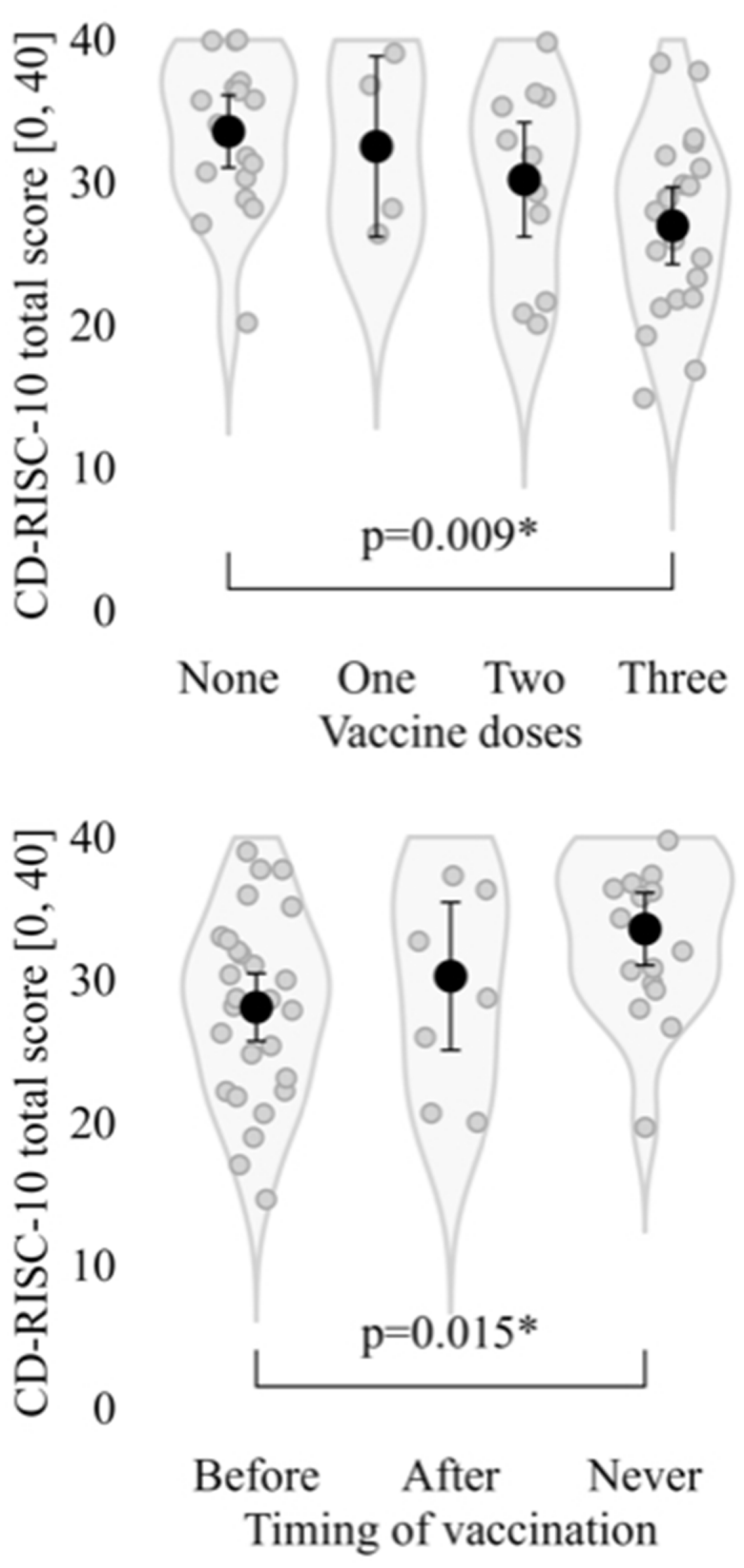
Total score at CD-RISC-10 by number of vaccine doses and timing of vaccination with reference to hospitalization. CD-RISC-10: Connor-Davidson’s Resilience Scale, 10-items. *: Statistically significant with *p*.

Results of the self-assessment tests are provided in Table 2. Non-vaccinated participants had higher total scores at the CD-RISC-10 than vaccinated ones (33.6 ± 5.50 vs. 28.6 ± 6.61; *t*_40.2_ = +2.94, *p* = 0.005), in particular they were less represented in the lower quartile [16.6% vs. 83.3%; OR = 0.235 (IC 95% 0.047, 0.943)], without statistically significant differences for distribution in other quartiles (all with *p* ≥ 0.273; Table 3). Instead, no statistically significant differences resulted between the two groups for having an internal locus of control [MLCS: OR = 1.488 (IC 95% 0.352, 6.024)], for cognitive status (I-TIC: t_47.8_ = +0.68, *p* = 0.498), for having a dysfunctional family [Family-Apgar: OR = 1.488 (IC 95% 0.352, 6.024)], for illness perception (Brief-IPQ total score: t_37.0_ = +0.06, *p* = 0.954; with *p* ≥ 0.091, for single items), and for presenting with psycho-traumatic symptoms (GPS symptoms: t_33.8_ = −0.55, *p* = 0.587; with *p* ≥ 0.083, for sub-scales and specific symptoms). Going into more detail (see Figure 1), the number of vaccine doses was significantly associated to the total CD- RISC-10 score (*F*_3,50_ = 3.91, *p* = 0.014), lowering from those who had not been vaccinated (33.6 ± 5.50) to those who had received three doses [27.0 ± 6.33; in post-hoc: Δ = +6.6 (+1.3, +11.9), *p* = 0.009]. Consistently, considering the distinction between participants who were vaccinated before hospitalization (i; CD-RISC-10: 28.1 ± 6.41), those vaccinated after hospitalization (ii; 30.3 ± 7.48), and those never vaccinated (iii; 33.6 ± 5.50), we observed a statistically significant difference (*F*_2,51_ = 4.17, *p* = 0.021), in particular between those vaccinated before hospitalization and those never vaccinated [Δ = +5.5 (+0.9, +10.1), *p* = 0.015].

**Table 2:** Sample description and vaccination status.

Test		% or Mean ± SD [min, Max]
CD-RISC-10	Total score	30.22 ± 6.652 [15.00, 40.00]
	Quartile of resilience	*1st quartile [0, 29]*: 44.4%
		*2nd quartile [30, 32]*: 16.7%
		*3rd quartile [33, 36]*: 20.4%
		*4th quartile [37, 40]*: 18.5%
MLCS	Classification	*Fatalist internalist*: 64.8%
		*Pure internalist*: 27.8%
		*Heterodependent fatalist*: 5.6%
		*Pure fatalist*: 1.9%
I-TIC	Total score	35.7 ± 5.32 [21, 48]
Family-Apgar	Classification	*Functional*: 84.4%
		*Moderately difunctional*: 11.1%
		*Severely dysfunctional*: 4.4%
Brief-IPQ	Total score	33.0 ± 15.14 [0, 68]
	1. Consequences	5.2 ± 3.57 [0, 10]
	2. Timeline	3.5 ± 3.33 [0, 10]
	3. Personal control^+^	5.5 ± 3.53 [0, 10]
	4. Therapy control^+^	6.7 ± 3.52 [0, 10]
	5. Intensity	3.3 ± 3.43 [0, 10]
	6. Concern	4.7 ± 3.48 [0, 10]
	7. Coherence^+^	5.3 ± 2.97 [0, 10]
	8. Emotions	3.8 ± 3.47 [0, 10]
GPS	Total symptoms	3.6 ± 2.95 [0, 12]
	PTSD	1.1 ± 1.28 [0, 5]
	CPTSD	1.5 ± 1.58 [0, 6]
	Risk	1.0 ± 1.17 [0, 4]
	DSO	0.4 ± 0.63 [0, 2]
	Anx	0.7 ± 0.65 [0, 2]
	Dep	0.6 ± 0.66 [0, 2]
	Ins	0.3 ± 0.46 [0, 1]
	SHI	0.0 [0, 0]
	Dis	0.1 ± 0.32 [0, 1]
	Sub	0.1 ± 0.23 [0, 1]
	Other problems	0.3 ± 0.44 [0, 1]

^+^: Higher scores are advantageous (Brief-IPQ); Anx, Anxiety items (GPS); Brief-IPQ: Brief Illness Perception Questionnaire; CD- RISC-10: Connor-Davidson’s Resilience Scale, 10-items; CPTSD: Complex Post-Traumatic Stress Disorder scale (GPS); Dep: Depression items (GPS); Dis: Dissociation items (GPS); DSO: Disturbances in Self-Organization items (GPS); Family-Apgar: Family Apgar; GPS: Global Psychodrama Screen, version 1.2; I-TIC: Telephone Interview for Cognitive Status, Italian version; Ins: Insomnia item (GPS); Max: Maximum observed value; min: minimum observed value; MLCS: Mini Locus of Control Scale; N: Number of observations; PTSD: Post-Traumatic Stress Disorder scale (GPS); Risk: Risks and protective factors scale (GPS); SD: Standard Deviation; SHI: Self-Harm Ideation item (GPS); Sub: Substance misuse item (GPS).

**Table 3:** Vaccination by quartile of resilience (CD-RISC-10 total score).

Quartile of Vaccination status (*in relation to hospitalization*)			
Resilience	*Before*	*After*	*Never*
1st, score: [0, 29]	66.7%	16.7%	16.7%
2nd, score: [30, 32]	55.6%	-	44.4%
3rd, score: [33, 36]	36.4%	18.2%	45.5%
4th, score: [37, 40]	30.0%	20.0%	50.0%

CD-RISC-10: Connor-Davidson’s Resilience Scale, 10-items.

### 3.3. Exploratory analyzes of resilience score predictors

**Figure 2:**
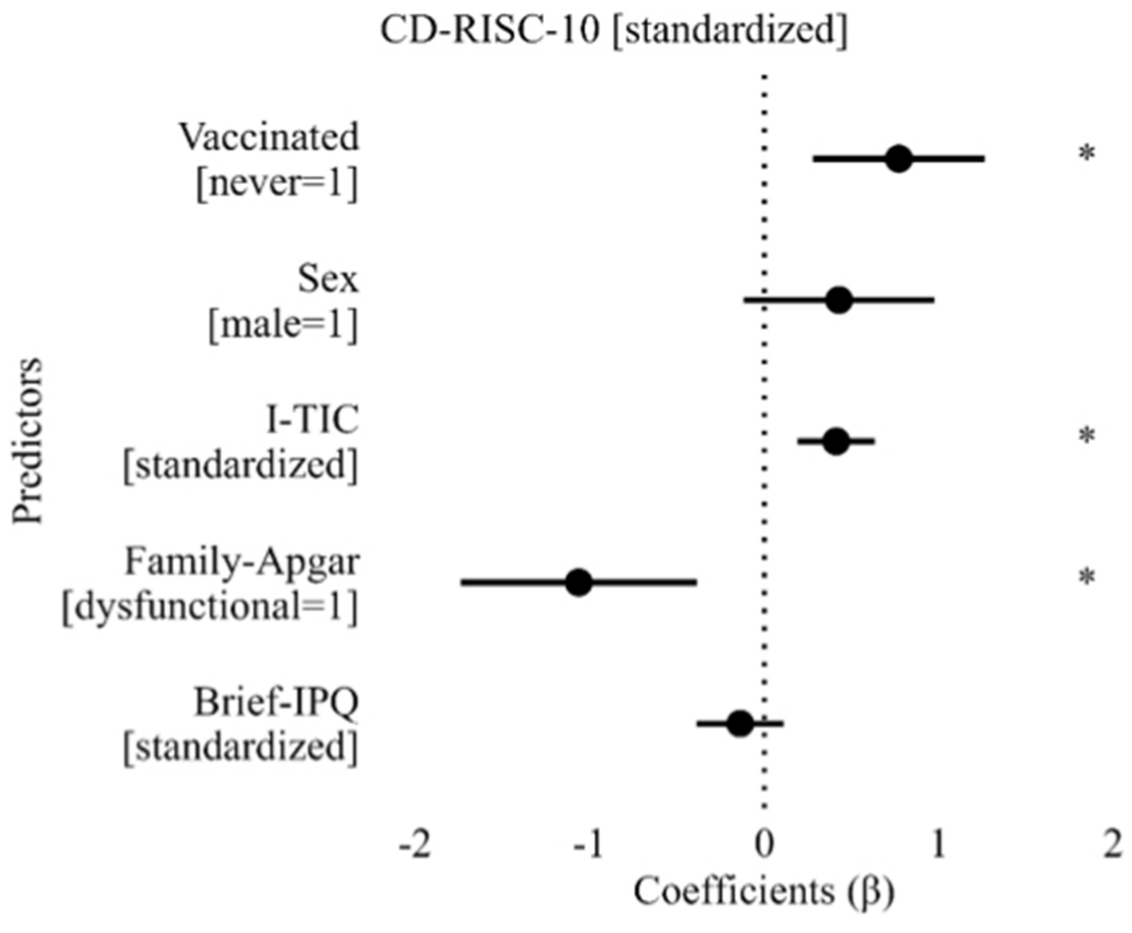
Results of multiple linear regression for CD-RISC-10 score. Brief-IPQ: Brief Illness Perception Questionnaire; CD-RISC-10: Connor-Davidson’s Resilience Scale, 10-items; Family-Apgar: Family Apgar; I-TIC: Telephone Interview for Cognitive Status, Italian version. *: Statistically significant predictor with *p*.

The CD-RISC-10 total score was not associated with age at hospitalization [*r* = −0.130 (−0.392, +0.151)], being single (*t*_44.4_ = +0.09), living alone (*t*_13.4_ = +1.04), having a low-level of schooling (*t*_28.3_ = +0.81), working (*t*_46.1_ = −1.77), having physical comorbidities (*t*_11.3_ = −1.42), being under psychopharmacological treatment (U = 126.0) and duration of hospitalization [*r* = −0.049 (−0.326, +0.235)]. Resilience was also not significantly associated with internal locus of control at the MLCS (*t*_33.5_ = −0.70) or symptoms at the GPS [*r* = −0.198 (−0.442, +0.074)]. Instead, males scored higher than females (31.4 ± 6.47 vs. 27.1 ± 6.30; *t*_26.1_ = −2.22) and the resilience score was found to be associated with the I-TIC score [*r* = +0.407 (+0.151, +0.612)], having a dysfunctional family according to the Family-Apgar questionnaire (*t*_7.5_ = +2.62) and the Brief-IPQ score [*r* = −0.293 (−0.520, −0.028)]. When statistically significant moderators were considered together (tolerance ranging between 0.819 and 0.976), the multiple regression was statistically significant (*R*^2^ = 0.568, adjusted to 0.510; *F*_5,37_ = 9.75). As can be seen in Figure 2, not being vaccinated was still shown to have a statistically significant association with resilience score (*β* = +0.771), cognitive status (I-TIC score: *β* = +0.412) and having a dysfunctional family (Family-Apgar: *β* = −1.062). The introduction of covariates reduced the β of vaccination by 7.9%.

## 4. Discussion

The COVID-19 global health emergency officially started on January 30th 2020 and ended on May 5th 2023. Incidence rates fluctuated over time and in Italy have been divided into four waves (20). The first wave happened during February–May 2020, the second during October–December 2020, the third during January–May 2021 and the fourth during November 2021–March 2022. The vaccination campaign started on December 27th 2020 and is still ongoing. This study has been conducted between the third and the fourth wave, when the percentage of vaccinated people increased from 24 to 80% (21), achieving the target population immunization rate (22). In the overall sample, 48.1% of patients were not vaccinated (i.e., with a ratio of 1.1 vaccinated persons for every non-vaccinated person), against an expectation of 15.9% (i.e., expected ratio: 5.3) (23). Such finding is in line with the higher risk of suffering from a form of COVID-19 requiring hospitalization among non-vaccinated individuals found in the relevant literature (24). A clinically significant higher mortality was also observed among non-vaccinated individuals (24). In a study conducted in 26 European countries in the same time period, the percentage of people declining COVID-19 vaccination was around 26% (25). Despite vaccination being among the key strategies implemented to limit the spread of the virus and improve health outcomes and life expectancy (26), a significant proportion of people have shown high levels of hesitancy. Vaccine hesitancy is a complex phenomenon, and it is influenced by several factors, including perceived need for the vaccine, accessibility of the vaccine, and perceived benefits and safety of the vaccine (25). Reduced vaccination intentions have been associated with female gender (27–33), high trust in media information sources (29, 34), low levels of trust in information from government sources, high conspiracy-mindedness (29), fear of side effects, and preference for a natural lifestyle (35). On the contrary, willingness to receive the vaccine has been associated with high education, high economic status, high perceived risk of infection (36–38), living with people with poor health, viewing vaccinations as a moral norm (39), positive attitude toward vaccines and previous vaccination (40), fear of COVID-19 and high levels of resilience (41). This study did not find any association between vaccination hesitancy and gender, economic status, and level of education. However, higher levels of resilience were found among non-vaccinated people, and such a result is worth of attention. Resilience refers to the process of bouncing back from difficult experiences and adapting well in the face of adversity, trauma, tragedy, threats, or significant sources of stress (42). It can be conceptualized at the individual (i.e., a stable trajectory of healthy functioning after a highly adverse event) (43), community (i.e., the success of the community to provide for the needs of its members and the extent to which individuals are helped by their community) (44), and societal (i.e., the perceived ability of the society to successfully deal with adversities and quickly recover after the threat has been removed) (45) levels. Individual resilience is closely related to identity resilience, that reflects the individual’s subjective belief in their capacity to understand and overcome challenges, their self-worth and value, their positive distinctiveness from others, and their certainty about who they have been and will remain (46). Identity resilience has two key components, which are *identity worth*, comprising self-efficacy, self-esteem, and positive distinctiveness, and *identity continuity*, depending upon feeling that the uniqueness and meaning of their identity persists over time (46). Regarding COVID-19, it has been found that identity worth is associated with less COVID-19 fear and less perceived COVID-19 risk, which could possibly result in a decrease in vaccination likelihood. One of the elements that define identity worth is self-esteem, and high levels of self-esteem have been associated with both healthy behaviors (47) and reduced probability of influenza vaccination (48). This has been explained by the tendency of individuals with high self-esteem to ignore disagreeable information and assume that calamities cannot happen, which can lead to declining vaccination (47). Thus, we can speculate that high levels of resilience among non-vaccinated people in our sample may be explained by high levels of identity worth and self-esteem.

It is worth mentioning that the high levels of vaccination hesitancy in our sample may result from the complex Italian sociopolitical situation during the pandemic. Italy was the first European country to be hit by the COVID-19 and since then several television personalities, politicians, media outlets, and even scientists have contributed to spreading conflicting and misleading information (49). This has generated a climate of uncertainty that has compromised trust in institutions, whose non- pharmacological interventions were often cataloged as exaggerated, and altered the risk perception of the population (50). The lack for trust in institutions has been related with an increase in vaccination hesitancy (51).

Limitations of this study include the single assessment performed during COVID-19-related hospitalization. In the absence of resilience information obtained outside of such a context, a confounding effect of hospitalization for COVID-19 cannot be ruled out. This may be particularly relevant for vaccinated individuals, whose resilience may have suffered a reduction for failing to avoid hospitalization despite having received one or more vaccine doses. Nevertheless, the current work may have important public health implications, as it indicates that specific psychological aspects, such as resilience, may modulate vaccination hesitancy, with implications for hospitalization rates, and thus healthcare costs, as well as loss of lives.

## 5. Conclusion

We carried out an observational study in order to explore psychological factors related to vaccination hesitancy in a sample of patients hospitalized for COVID-19 in a later phase of the pandemic when vaccines had been made fully available to the general population. We found higher resilience scores in non-vaccinated patients compared to vaccinated ones. This result may be explained by high levels of identity worth and self-esteem among non-vaccinated people, making them less prone to vaccination. Future studies will have to focus not only on psychological but also on broader socio-behavioral determinants of compliance with health and mental care recommendations (e.g., lack of trust in institutions), in order to better understand the vaccination hesitancy phenomenon.

## Data availability statement

The raw data supporting the conclusions of this article will be made available by the authors, without undue reservation.

## Ethics statement

The studies involving humans were approved by Unique Regional Ethics Commitee (CEUR-2021-OS-19). The studies were conducted in accordance with the local legislation and institutional requirements. The participants provided their written informed consent to participate in this study.

## Author contributions

CC: Conceptualization, Data curation, Methodology, Resources, Validation, Visualization, Writing – original draft, Writing – review & editing. MCe: Conceptualization, Data curation, Investigation, Resources, Validation, Visualization, Writing – original draft, Writing – review & editing. RM: Conceptualization, Data curation, Investigation, Resources, Validation, Visualization, Writing – original draft, Writing – review & editing. MG: Conceptualization, Data curation, Formal analysis, Methodology, Resources, Validation, Visualization, Writing – original draft, Writing – review & editing. RB: Conceptualization, Data curation, Investigation, Validation, Visualization, Writing – review & editing. NI: Conceptualization, Data curation, Investigation, Resources, Validation, Visualization, Writing – review & editing. ES: Conceptualization, Resources, Validation, Visualization, Writing – review & editing. CT: Conceptualization, Resources, Supervision, Validation, Visualization, Writing – review & editing. MB: Conceptualization, Data curation, Methodology, Project administration, Resources, Supervision, Validation, Visualization, Writing – review & editing. MCo: Conceptualization, Data curation, Methodology, Project administration, Resources, Supervision, Validation, Visualization, Writing – original draft, Writing – review & editing.
